# Parental Misperception of Child’s Weight: A Barrier to Combating the Childhood Obesity Epidemic in a Lower Middle-Income Country

**DOI:** 10.1192/j.eurpsy.2026.11531

**Published:** 2026-07-31

**Authors:** R. Ghammam, R. Kebaili, S. Ben Fredj, N. Zammit, A. Skhiri, O. K. Ben Saad, H. Ben Hafaiedh, N. Jaballah, F. Chouikha, A. Chouchene, L. Boughammoura, I. Harrabi, M. Jihene

**Affiliations:** 1 Université de Sousse, Faculté de Médecine de Sousse; 2 Hôpital Universitaire Farhat Hached, Service de Médecine Préventive et Communautaire, LR19SP03; 3 Service de Pédiatrie, CHU Farhat Hached, Université de Sousse, Faculté de Médecine de Sousse; 4 Hôpital Universitaier Farhat HachedService de Pédiatrie,; 5 Médecie de Famille, Université de Sousse, Faculté de Médecine de Sousse; 6 Médecine Préventive et Communautaire, Hopital Universitaire Farhat hached; 7 Hôpital Universitaire Farhat Hached, Service de Médecine de Travail, LR19SP03, Sousse, Tunisia

## Abstract

**Introduction:**

The global obesity pandemic poses a significant threat to public health. A critical first step in addressing childhood obesity is ensuring that parents can accurately identify their child’s weight status. However, a widespread misperception of a child’s weight, particularly an underestimation of overweight or obesity, is a major barrier to effective prevention and intervention. Understanding the factors that contribute to this misperception is essential for developing targeted public health strategies.

**Objectives:**

The objective of this study was to evaluate parental perception of their child’s weight and investigate the factors associated with underestimation in a lower middle-income country (LMIC).

**Methods:**

This cross-sectional study was conducted in six randomly selected kindergartens to ensure a representative sample. All enrolled children and their parents were invited to participate. Data were collected via a pre-tested, self-administered questionnaire in Arabic. Anthropometric measurements were taken for all participating children

**Results:**

A total of 364 children aged 3 to 5 years, along with their parents, participated. Of the children, 51.4% were boys. A striking 63.1% (95% CI [58.1-68.1]) of parents underestimated their child’s weight, while only 34.9% (95% CI [29.9-39.8]) perceived it correctly. The remaining 2% (95% CI [0.5-3.3]) overestimated it.

Multivariate analysis showed a strong link between parental underestimation of a child’s weight and several key factors. We found that parents who underestimated their own weight were over six times more likely to underestimate their child’s weight (aOR=6.43, 95% CI [2.48-16.66], p<0.001). Additionally, a father’s excess weight (aOR=3.22, 95% CI [1.53-6.77], p=0.002) and a child’s overweight status (aOR=6.01, 95% CI [1.16-31.14], p=0.033) were also significantly associated with this misperception. A similar trend was observed for childhood obesity (OR=3.59, 95% CI [0.93-13.87], p=0.063).

**Conclusions:**

Our findings reveal that a significant majority of parents in this LMIC setting underestimate their child’s weight. This widespread misperception is a critical barrier to effective obesity prevention and may play a crucial role in sustaining the obesity epidemic in developing countries. To address this, a multidisciplinary strategy is urgently needed to not only modify lifestyle and environmental factors but also to change cultural and social norms surrounding weight perception.

**Disclosure of Interest:**

None Declared

